# Analysis of Insulin Resistance Among Children and Adolescents in Slovenia With Hypercholesterolemia After Treatment With Statins

**DOI:** 10.1001/jamanetworkopen.2022.31097

**Published:** 2022-09-12

**Authors:** Urh Groselj, Jaka Sikonja, Matej Mlinaric, Primoz Kotnik, Tadej Battelino, Joshua W. Knowles

**Affiliations:** 1 Department of Endocrinology, Diabetes, and Metabolic Diseases, University Children’s Hospital, University Medical Center Ljubljana, Ljubljana, Slovenia; 2 Division of Cardiovascular Medicine, Department of Medicine, Stanford University School of Medicine, Stanford, California

## Abstract

This cohort study assesses the association between short-term treatment with rosuvastatin therapy and changes in insulin resistance markers among children and adolescents in Slovenia who have hypercholesterolemia.

## Introduction

Statins are the first-line treatment for lowering atherogenic low-density lipoprotein cholesterol (LDL-C) in children with familial hypercholesterolemia (FH).^1,2^ Clinical trials have demonstrated the efficacy, safety, and good tolerability of statins among children with FH.^3^ The number of adolescents with FH needed to treat to prevent 1 heart attack is approximately 2.^4^ In adults, statins are associated with increased insulin resistance, insulin secretion, and type 2 diabetes risk.^5,6^ Data on the effects of statins on glucose homeostasis are lacking for children, who require lifelong lipid-lowering treatment (LLT).^1,2,4^ We assessed the association between treatment with statins and changes in insulin resistance markers in children and adolescents in Slovenia.

## Methods

The National Medical Ethics Committee of Slovenia approved this cohort study. Written informed consent was obtained from participants or their parents/caregivers. The study followed the STROBE reporting guideline.

Children and adolescents diagnosed with FH were included if they met the criteria for LLT initiation^1^ and had fasting insulin and glucose measurements for 2 consecutive visits between 2016 and 2021. The treatment group comprised individuals who were prescribed 5 mg of rosuvastatin at baseline, had unmodified therapy between consecutive visits, and had a 20% or greater reduction in LDL-C from baseline. Control participants were those receiving no therapy despite intention to treat. Race and ethnicity were self-reported by participants or their parents/caregivers. Median values are reported with IQRs. Nonparametric statistical tests (all 2-tailed) were used; *P* < 0.05 was considered significant. Data were analyzed using IBM SPSS Statistics, version 26.0 (IBM).

## Results

This study included 35 participants: 20 in the treatment group (8 girls [40.0%], 12 boys [60.0%]; median [IQR] age at baseline, 10.0 [8.7-11.6] years) and 15 in the control group (7 girls [46.7%], 8 boys [53.3%]; median [IQR] age at baseline, 10.2 [8.8-13.2] years). Eighteen treatment group (90.0%) and 9 control group (60.0%) participants had a positive genetic test result for FH. All participants were White. Treatment and control group follow-up visits were performed at a median of 7.4 (6.4-12.6) and 13.4 (11.5-19.6) months (*P* = .03) from baseline, respectively.

Although median baseline LDL-C was higher among treatment group vs control group participants (209 [189-267] vs 174 [160-193] mg/dL [to convert to millimoles per liter, multiply by 0.0259]; *P* = .01), glucose homeostasis parameters at baseline were comparable (Table). Participants taking rosuvastatin had median reductions of 40.2% (34.6%-47.0%) in LDL-C (*P* < .001) (Figure) and 32.6% (24.9%-39.2%) in total cholesterol (*P* < .001). No changes in LDL-C and total cholesterol were observed among control participants; end-of-study LDL-C levels were lower in the treatment group (Table). No changes in median body mass index *z* scores were observed among treatment or control participants. Rosuvastatin was not associated with increased fasting glucose, fasting insulin, or HOMA-IR (Table). HbA_1c_ levels remained unchanged.

**Table.** Changes in Cholesterol and Indirect Parameters of Insulin Resistance Among the Study Cohort

Marker	Rosuvastatin, 5 mg (n = 20)				Control (n = 15)			
	Median (IQR)			*P* value^a^	Median (IQR)			*P* value
	Baseline	Follow-up	Between-visit difference		Baseline	Follow-up	Between-visit difference	
LDL-C, mg/dL	209 (189-267)	124 (112-144)	−77 (−105 to −69)	<.001	174 (160-193)	170 (164-188)	−4 (−21 to 8)	.21
Total cholesterol, mg/dL	292 (266-341)	191 (177-219)	−87 (−122 to −70)	<.001	255 (234-264)	247 (236-257)	−12 (−15 to 2)	.16
Insulin, μIU/mL	7.6 (3.2-11.0)	7.2 (4.8-12.0)	0 (−3.5 to 3.3)	.87	3.3 (2.4-13.9)	5.9 (3.2-12)	0 (−4.4 to 2.2)	.93
Glucose, mg/dL	84 (80-87)	83 (81-85)	0 (−2 to 4)	.90	79 (77-83)	83 (78-87)	4 (−2 to 5)	.35
HOMA-IR	1.5 (0.63-2.1)	1.4 (0.9-2.5)	0.1 (−0.8 to 0.7)	.97	0.75 (0.47-2.80)	1.1 (0.62-2.4)	0 (−0.8 to 0.5)	>.99
HbA_1c_, %	5.3 (5.2-5.4)	5.2 (5.1-5.4)	0 (−0.2 to 0.1)	.46	5.2 (5.1-5.3)	5.3 (5.0-5.3)	0.1 (−0.1 to 0.2)	.44

Abbreviations: HbA_1c_, hemoglobin A_1c_; HOMA-IR, homeostasis model assessment of insulin resistance index; LDL-C, low-density lipoprotein cholesterol.

^a^ *P* values were calculated for between-visit differences among groups. SI conversion factors: To convert LDL-C to millimoles per liter, multiply by 0.0259; to convert total cholesterol to picomoles per liter, multiply by 6.945; to convert glucose to millimoles per liter, multiply by 0.0555; and to convert HbA_1c_ to proportion of total hemoglobin, multiply by 0.01.

**Figure.**
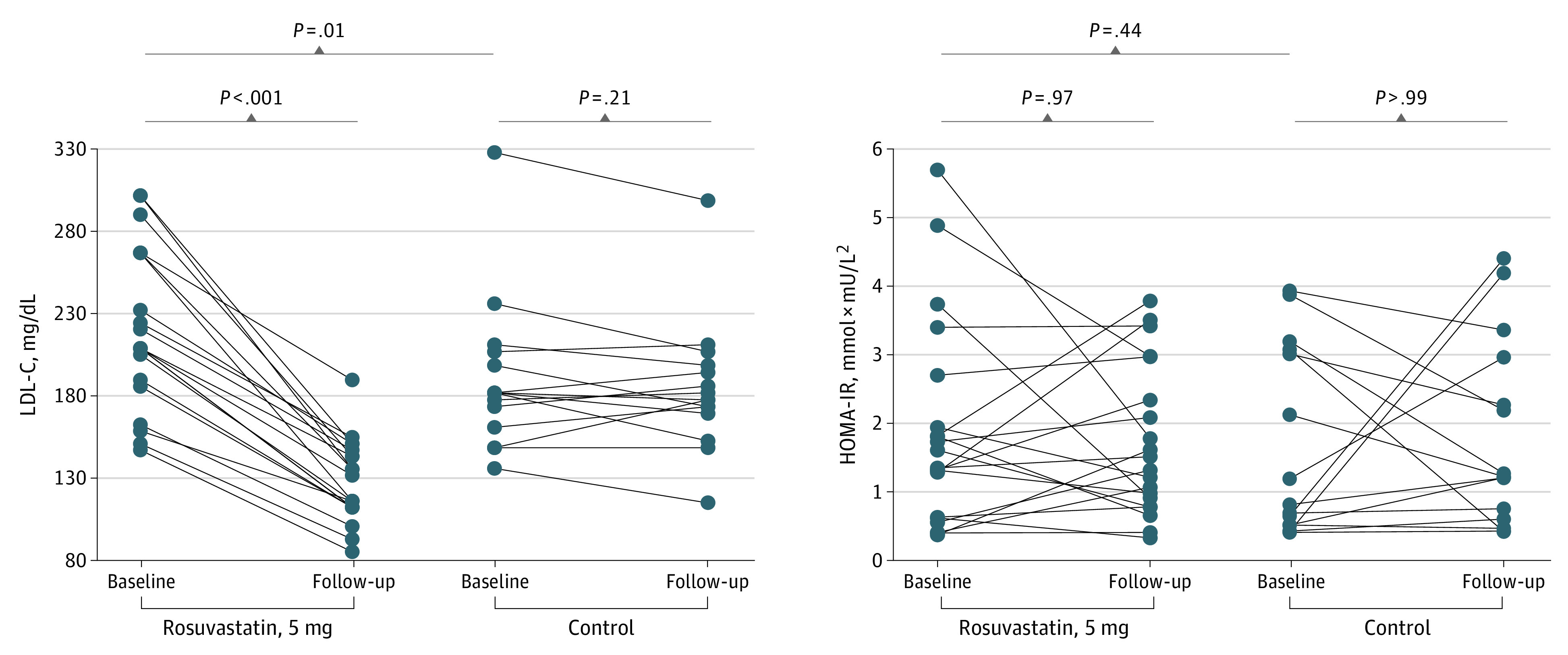
Association of Rosuvastatin Treatment and Nontreatment With Changes in Insulin Markers Among the Study Cohort HOMA-IR indicates homeostasis model assessment of insulin resistance index; LDL-C, low-density lipoprotein cholesterol. To convert LDL-C to millimoles per liter, multiply by 0.0259.

## Discussion

Children with FH who sustain a lifelong reduction in LDL-C with LLT have a lower risk of cardiovascular disease.^1,2,4^ In this cohort study, substantial reductions in LDL-C among children and adolescents taking rosuvastatin were not accompanied by increased fasting glucose, fasting insulin, HOMA-IR, and HbA_1c_ levels after 7 months. In contrast, studies of adults suggest that changes can be observed 10 weeks after statin initiation.^6^

Study limitations include the small sample size, short follow-up, low rosuvastatin dose, and lack of data on family history and pubertal status. Short-term treatment with rosuvastatin was not associated with changes in insulin resistance markers in this cohort of children with FH.
